# Optical coherency matrix tomography

**DOI:** 10.1038/srep15333

**Published:** 2015-10-19

**Authors:** Kumel H. Kagalwala, H. Esat Kondakci, Ayman F. Abouraddy, Bahaa E. A. Saleh

**Affiliations:** 1 CREOL, The College of Optics & Photonics, University of Central Florida, Orlando, Florida 32816, USA

## Abstract

The coherence of an optical beam having multiple degrees of freedom (DoFs) is described by a coherency matrix **G** spanning these DoFs. This optical coherency matrix has not been measured in its entirety to date—even in the simplest case of two binary DoFs where **G** is a 4 × 4 matrix. We establish a methodical yet versatile approach—optical coherency matrix tomography—for reconstructing **G** that exploits the analogy between this problem in classical optics and that of tomographically reconstructing the density matrix associated with multipartite quantum states in quantum information science. Here **G** is reconstructed from a minimal set of linearly independent measurements, each a cascade of projective measurements for each DoF. We report the first experimental measurements of the 4 × 4 coherency matrix **G** associated with an electromagnetic beam in which polarization and a spatial DoF are relevant, ranging from the traditional two-point Young’s double slit to spatial parity and orbital angular momentum modes.

The statistical fluctuations of light in space and time may be characterized by a hierarchy of correlation functions for electromagnetic field components^1^,^2^. These functions, not the optical fields themselves, provide a description of light in terms of observable quantities^3^. The theory of optical coherence investigates the properties of these correlation functions pertaining to the temporal, spatial, and polarization degrees of freedom (DoFs). When these DoFs are uncoupled (or uncorrelated), simple measures of coherence for *each* DoF suffice, such as coherence time, coherence area, and degree of polarization^4^. However, when the DoFs are *coupled*, such measures lose their utility and more sophisticated approaches are required, such as the mutual coherence function^5^, the beam coherence-polarization matrix^6^,^7^,^8^, or the 4 × 4 field correlation matrix for a pair of points in an electromagnetic field^9^,^10^,^11^.

While the importance of coupling between DoFs was recognized decades ago, as in Mandel’s seminal work on optical cross-spectral purity (the absence of spatial-spectral coupling)^12^, recent advances have led to a host of scenarios wherein such coupling is critical. For example, vector beams correlate polarization with spatial position^13^, scattering from complex photonic structures and devices may couple the relevant field DoFs^14^,^15^, and reliance on multimode optical fibers for spatial multiplexing is reviving interest in joint polarization-spatial-mode characterization^16^. In exploring these settings, it has recently proven fruitful to adopt the Hilbert-space formulation used in quantum mechanics to the needs of classical coherence theory^10^,^11^—an approach that has early prescient antecedents^17^,^18^. In the context of coupling between multiple DoFs, such a treatment necessitates introducing the notion of ‘classical entanglement’^10^,^19^,^20^,^21^,^22^,^23^,^24^,^25^. In quantum mechanics, states associated with bipartite systems that do not separate into products of states belonging to the Hilbert space of each particle are said to be *entangled*^26^. As a consequence of the mathematical similarity between the Hilbert spaces of multi-partite quantum states and multi-DoF classical optical fields, a corresponding concept of *classical* entanglement indicates the non-separability of the beam into uncoupled DoFs. After the initial suggestion by Spreeuw^19^, a substantial body of work has accumulated in the past five years in which classical entanglement is exploited in solving long-standing problems in polarization optics^27^,^28^,^29^, delineating the contributions of non-separability and intrinsic randomness to the coherence of an optical beam^10^,^30^, introducing new metrology schemes^31^, and implementing classical analogs of quantum information processing protocols, such as teleportation^32^,^33^, and super-dense coding, etc.

A fundamental capability that has remained elusive in classical optics is the complete identification of the coherence function for a beam with coupled DoFs. In quantum mechanics, the task of measuring all the elements of a density matrix is known as ‘quantum state tomography’^34^,^35^. The corresponding procedure for multi-DoF beams in classical optics has been studied theoretically^11^, but has not been demonstrated experimentally heretofore. Even in the simplest case of two binary DoFs^6^ (e.g., polarization, a bimodal waveguide^36^,^37^, two coupled single-mode waveguides^38^,^39^, spatial-parity modes^40^,^41^,^42^,^43^,^44^, etc.), the associated 4 × 4 coherency matrix **G**, which is a complete representation of second-order coherence^10^,^11^, has *not* been measured in its entirety to date.

## Results

In this Article, we present a methodical approach—optical coherency *matrix* tomography (OC*m*T)—for measuring the complex elements of 4 × 4 coherency matrices **G** by appropriating the quantum-state-tomography strategy. To demonstrate the universality of our approach, we implement it with coherent and partially coherent fields having coupled or uncoupled DoFs in three distinct settings involving pairs of points^9^,^10^,^11^, spatial-parity modes^40^,^41^,^42^,^43^,^44^, and orbital angular momentum (OAM) modes^45^—each together with polarization. We identify the minimal set of linearly independent, joint spatial-polarization projective measurements that enable a unique reconstruction of **G**. Since **G** is a complete representation of the field, its reconstruction obviates the need to measure directly any coherence descriptors (all of which are scalar functions of the complex elements of **G**) and, moreover, allows for unambiguous identification of classical entanglement.

The coherence of an optical beam having a single binary DoF is represented by a 2 × 2 Hermitian coherency matrix 
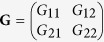
, where 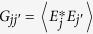, 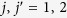, and *E*_*j*_ is the field corresponding to one *level* of the DoF. For example, polarization is represented by the coherency matrix^4^


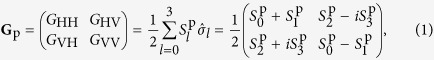


where 
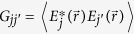
, 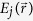 is the horizontal (H) or vertical (V) field component at a point 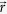, 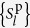 are the Stokes parameters, and 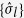 are the Pauli matrices


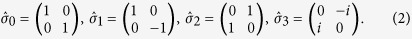


**Figure 1:**
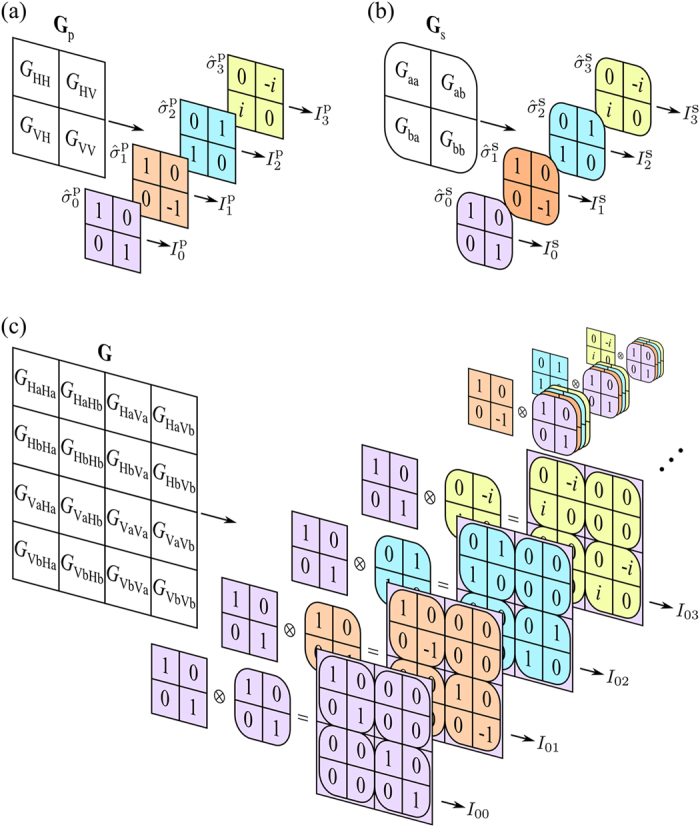
Measurement scheme for optical coherency *matrix* tomography (OC*m*T). (**a**) Four projections 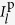, 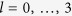 to obtain the Stokes parameters {*S*_*l*_} and reconstruct **G**_p_ for the polarization DoF. (**b**) Similarly, four projections 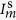, 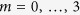 to obtain the Stokes parameters {*S*_*m*_} and reconstruct **G**_s_ for a binary spatial DoF. (**c**) OC*m*T enables the reconstruction of **G** for the two binary DoFs in (**a**,**b**) considered simultaneously via 16 joint polarization-spatial measurements, each of which consists of a cascade of two projections—one from (**a**) and the other from (**b**).

Polarization coherence is quantified by the degree of polarization 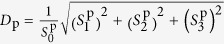
—with 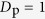 and 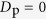 corresponding to purely polarized and completely unpolarized light, respectively. The Stokes parameters are evaluated via four projective polarization measurements [Fig. 1(a)]: 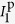, 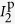, and 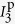 correspond to the H, diagonal (D), and right-hand-circular (R) polarization components, respectively, in addition to the total power 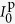 ref. 46; in which case 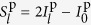. The *same* formalism may be applied to *other* binary DoFs [Fig. 1(b)]: (i) the scalar field at two points 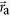 and 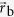, *E*_a_ and *E*_b_; (ii) the spatial-parity even ‘e’ and odd ‘o’ modes of a scalar field *E*_e_ and *E*_o_ refs. 40, 41, 42, 43, 44; or (iii) a pair of OAM modes, e.g., *E*_0_ and *E*_1_ corresponding to OAM 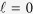 and 1, respectively^45^,^47^.

When two binary DoFs of the field are relevant, e.g., the first is polarization ‘p’ and the second is a spatial ‘s’ DoF with modes identified as ‘a’ and ‘b’ [Fig. 1(c)]—the corresponding coherency matrix **G** is now 4 × 4 refs. 10, 11,


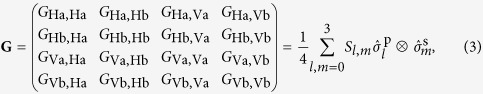


where 
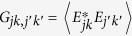
, 
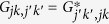
, *E*_*jk*_ is a field component with *j* = H, V and *k* = a, b, {*S*_*lm*_} are the two-DoF Stokes parameters, and 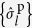 and 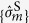 are the Pauli matrices on the polarization- and spatial-DoF Hilbert subspaces, respectively^11^. In determining the coherence descriptors of each DoF independently of the other, one first traces over the other DoF to obtain a *reduced* coherency matrix^10^. The reduced *polarization* coherency matrix **G**_p_, obtained by tracing over the *spatial* DoF in **G**, is given by


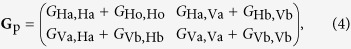


while the reduced coherency matrix for the *spatial* DoF **G**_s_, obtained after tracing over *polarization* in **G** (see ref. 11), is given by


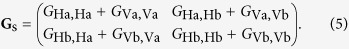


The elements of the reduced coherency matrices are measured by a system sensitive to one DoF, but *not* to the other. When the two DoFs are uncoupled, then 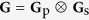, otherwise the elements of **G**_p_ and **G**_s_ lack information about the correlations between the two DoFs that is contained in **G**. Such correlations are only measurable by a system that is sensitive to both DoFs via joint polarization-spatial measurements.

We pose the following question: what are the necessary and sufficient measurements to reconstruct an arbitrary **G** for two binary DoFs? This question was solved by Wootters^48^ in the context of reconstructing the density matrix 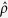 for a bipartite quantum system. He showed that the measurements carried out on each subsystem to reconstruct its reduced density matrix are sufficient to reconstruct 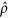 when carried out *jointly*—a methodology known as quantum state tomography^34^,^35^. In our context of a classical optical beam having two binary DoFs, the analogy with the quantum setting allows us to exploit the same strategy. Regardless of the specific form of **G**, the necessary measurements to carry out OC*m*T and reconstruct **G** [Fig. 1(c)] are those used to reconstruct the reduced coherency matrices [Fig. 1(a,b)] carried out in cascades of pairs of projections—one for polarization and the other for the spatial DoF. Each measurement yields a real number *I*_*lm*_ (projection *l* for polarization and *m* for the spatial DoF) corresponding to the projection of a tomographic slice through **G**. The 16 combinations of polarization-spatial measurements are inverted to obtain the two-DoF Stokes parameters, 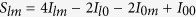, and hence reconstruct **G** (see ref. 11 for details).

We have performed a series of experiments implementing the OC*m*T scheme described above using quasi-monochromatic beams having two binary DoFs: polarization and a spatial DoF. We have measured the 4 × 4 coherency matrix **G** for six different beams corresponding to distinct states of light having the following properties:

**G**_1_: the polarization and spatial DoFs are separable and both are coherent.

**G**_2_: the polarization DoF is coherent while the spatial DoF lacks coherence.

**G**_3_: both the polarization and spatial DoFs lack coherence.

**G**_4_: the polarization and spatial DoFs are classically entangled.

**G**_5_: the polarization and spatial DoFs are classically correlated.

**G**_6_: this beam is a mixture of the separable-coherent beam **G**_1_ and the classically entangled beam **G**_4_.

We use the sequence of polarization projections described earlier and present below the spatial projections following the H projection (similar spatial projections are carried out following the V, D and R polarization projections).

### Polarization with Spatial Position

**Figure 2:**
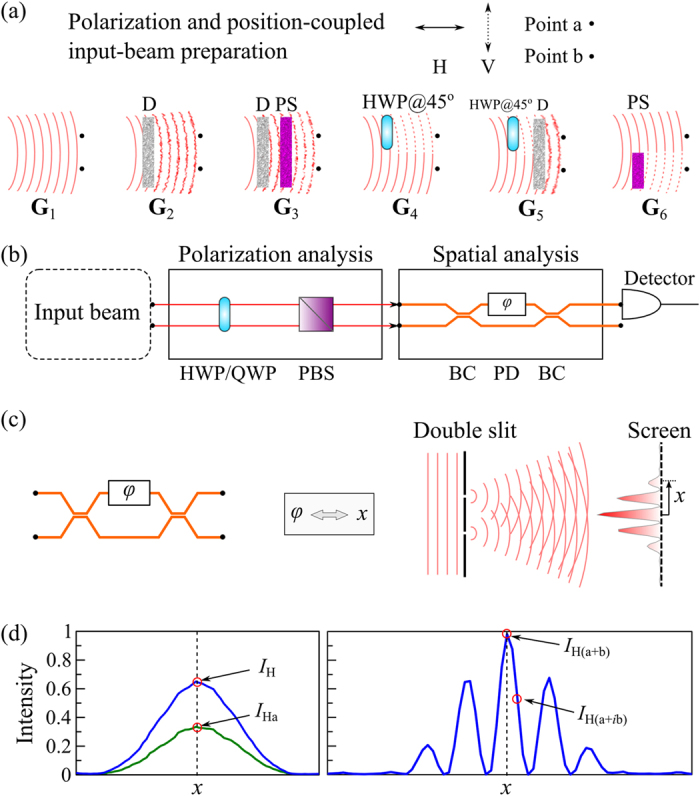
Polarization considered with spatial location in a Young’s double-slit configuration. (**a**) Experimental setups for preparing polarization and position-coupled beams **G**_1_ through **G**_6_; D: diffuser that randomizes the beam spatially; PS: polarization scrambler that randomizes the beam polarization; HWP: half-wave plate. (**b**) Experimental setup delineating the stages of polarization analysis followed by spatial analysis. HWP: half-wave plate; QWP: quarter-wave plate; PBS: polarizing beam splitter; BC: 50:50 beam coupler; PD: phase delay element that introduces a phase shift 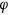. (**c**) Illustration depicting the equivalence between the phase shift 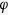 introduced by the phase element PD, and the lateral displacement *x* on a screen upon which the far-field intensity pattern is projected. (**d**) Spatial profile measurements obtained by a CCD camera illustrating the spatial projective measurements for the H polarization projection; similarly for the V, D, and R projections.

The first realization of the spatial DoF is the traditional two points, as in the Young’s double slit experiment. The polarization and position-coupled beam is prepared in one of six states **G**_1_ through **G**_6_; Fig. 2(a) (see Supplementary information for details). OC*m*T for a such a beam comprises of the polarization analysis followed by the spatial analysis; Fig. 2(b). The spatial analysis may alternatively be carried out by extracting specific intensity points from the far-field intensity patterns for only two values of displacement *x* on a screen or an array of detectors; Fig. 2(c). The four spatial projections are obtained by measuring the following: (1) the total power from both points *I*_10_ = *I*_H_ at *x* = 0; (2) the power from point ‘a’ *I*_11_ = *I*_Ha_ at *x* = 0; (3) the power in the far-field interference pattern 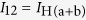 at *x* = 0; and (4) the power at the value of *x* corresponding to a 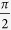 phase shift 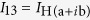; see Fig. 2(d). It is important to note that the visibility of fringes is not the parameter sought here to characterize the spatial coherence at ‘a’ and ‘b’; instead the four points identified in Fig. 2(d), together with the set of points obtained for the V, D, and R polarization projections, reveal the complete picture even when polarization and the spatial DoFs are classically entangled.

### Polarization with Spatial Parity

**Figure 3:**
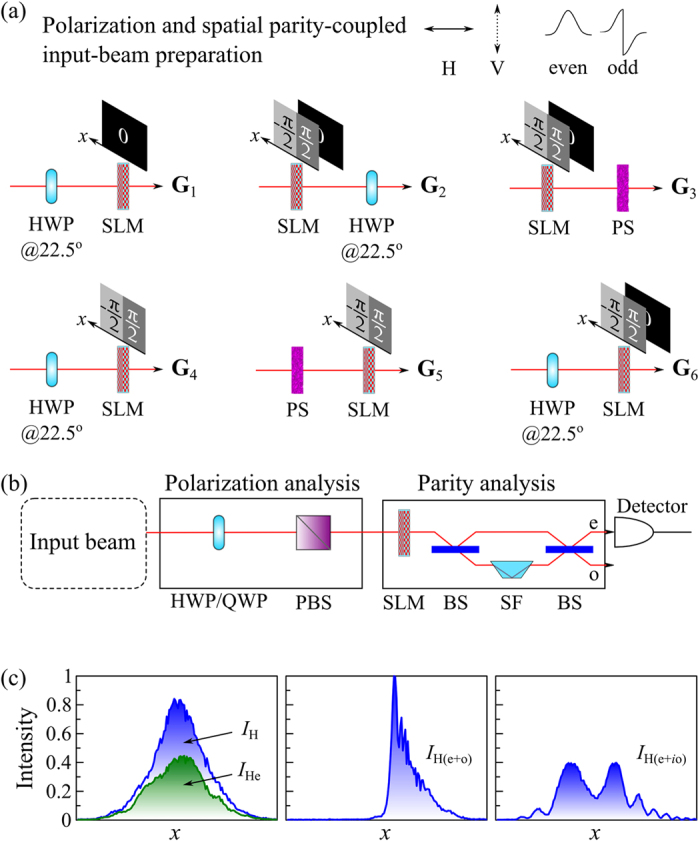
Polarization considered with spatial parity modes. (**a**) Experimental setups for preparing polarization and spatial-parity-coupled beams **G**_1_ through **G**_6_; HWP: half-wave plate; SLM: spatial light modulator; PS: polarization scrambler that randomizes the beam polarization. (**b**) Experimental setup delineating the stages of polarization analysis followed by spatial-parity analysis. HWP: half-wave plate; QWP: quarter-wave plate; PBS: polarizing beam splitter; SLM: spatial light modulator; BS: beam splitter; SF: spatial flipper that flips the sign of the odd ‘o’ spatial-parity mode, leaving the even ‘e’ mode intact. (**c**) Corresponding spatial profile measurements obtained by a CCD camera illustrating the spatial projective measurements for the H polarization projection; similarly for the V, D, and R projections.

The second spatial-DoF realization makes use of one-dimensional even ‘e’ and odd ‘o’ spatial-parity modes with respect to *x* = 0. The polarization and spatial parity-coupled beam is prepared in one of six states **G**_1_ through **G**_6_; Fig. 3(a) (see Supplementary information for details). OC*m*T for a such a beam comprises of the polarization analysis followed by the spatial-parity analysis; Fig. 3(b). The four spatial projections are obtained by measuring the power (integrated over the shaded areas in Fig. 3(c)) in the following settings: (1) the total power *I*_10_ = *I*_H_ of the beam; (2) the power of the even component *I*_11_ = *I*_He_ obtained from a modified Mach-Zehnder interferometer that separates the beam into the different spatial-parity components^41^; (3) the power 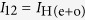 after blocking the half-plane *x* < 0, corresponding to a projection onto the 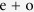 component; and (4) a projection onto the 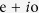 component obtained from the power 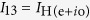 of the even component measured after first introducing a phase-step 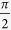 between the two plane halves *x* < 0 and *x* ≥ 0 implemented by a spatial light modulator (SLM); see Fig. 3(c). This phase modulation was shown in ref. 40, 41, 42, 43, 44 to produce a rotation on a major circle on a Poincaré sphere having the e and o modes as antipodes.

### Polarization with OAM

**Figure 4:**
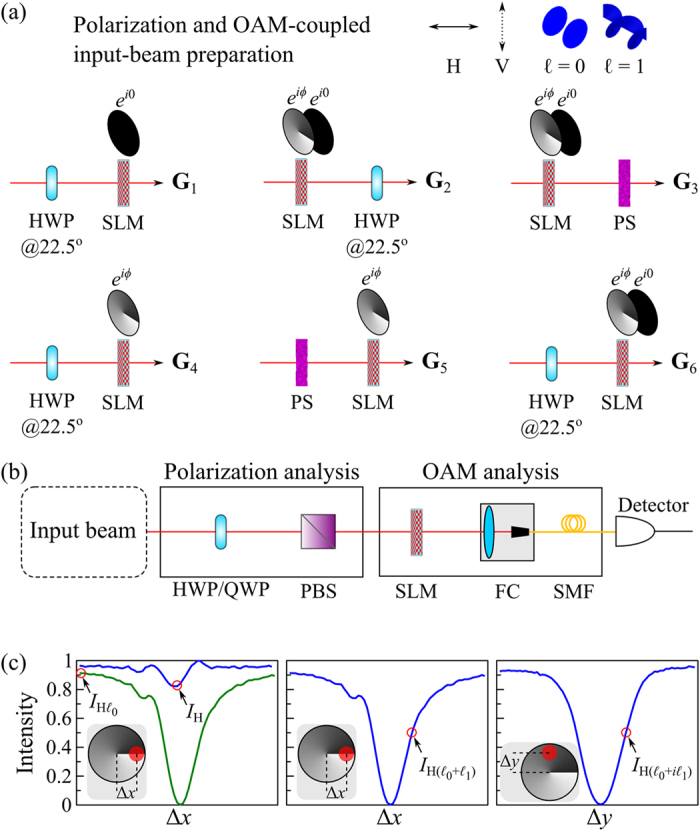
Polarization considered with OAM modes. (**a**) Experimental setups for preparing polarization and OAM-coupled beams **G**_1_ through **G**_6_; HWP: half-wave plate; SLM: spatial light modulator; PS: polarization scrambler that randomizes the beam polarization. (**b**) Experimental setup delineating the stages of polarization analysis followed by OAM-mode analysis. HWP: half-wave plate; QWP: quarter-wave plate; PBS: polarizing beam splitter; SLM: spatial light modulator; FC: fiber coupler; SMF: single-mode fiber. (**c**) Corresponding spatial profile measurements obtained by a power-meter illustrating the spatial projective measurements for the H polarization projection; similarly for the V, D, and R projections. Spatial measurements are obtained by dislocating the phase singularity 
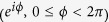
 relative to the beam Δ*x* along *x* and Δ*y* along *y*. *I*_H_ is obtained by adding the intensities 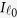 at 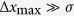 (*σ* is the beam width) and 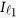 at Δ*x* = 0, 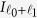 is obtained at 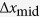, and 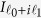 is obtained at 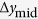 (Δ*x*_mid_ and Δ*y*_mid_ are calibrated using a Gaussian beam).

The third realization exploits two low-order OAM modes 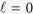 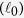 and 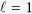 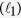. The polarization and OAM-coupled beam is prepared in one of six states **G**_1_ through **G**_6_; Fig. 4(a) (see Supplementary information for details). OC*m*T for a such a beam comprises of the polarization analysis followed by the OAM mode analysis; Fig. 4(b).The four spatial projections are obtained by measuring the following: (1) the total power *I*_10_ = *I*_H_; (2) the power of the 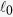 component 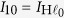 obtained using a spatial filter (a lens focusing to a single-mode fiber); (3) the power of the 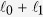 component using a phase vortex with the dislocation displaced laterally with respect to the beam implemented using an SLM 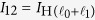 ref. 49; and (4) the power of the 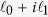 component using the same phase vortex but with the dislocation displaced vertically 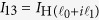; see Fig. 4(c).

### Measurements

We have measured the complex elements of **G** for six different classes of beams comprising those with separable DoFs (both coherent, both incoherent, or in a hybrid coherent/incoherent configuration), non-separable DoFs (classically entangled or classically correlated), and mixtures of beams from the two separable and non-separable classes (see Supplementary information for the complete results). In each experiment, the prepared beam passes first through polarization then spatial-DoF analysis stages (the order may be reversed without changing the outcome). In each of these realizations, permutations of the four polarization projection settings combined with the four spatial projection settings yield 16 measurements for OC*m*T, which are used to reconstruct **G**. We make use of a maximal-likelihood algorithm that exploits the constraints set by the trace, hermiticity, and semi-positive-definiteness of **G**^50^. We portray the real and imaginary components of **G** using the standard visualization from quantum state tomography. In each plot we provide the coherence descriptor for the polarization *D*_p_ and spatial DoF *D*_s_ obtained from their reduced coherency matrices, in addition to the linear entropy 
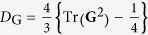
, which serves as a measure of the overall beam coherence, where *D*_G_ ranges from 0 (complete incoherence in all DoFs) to 1 (a coherent beam with no statistical fluctuations)^51^. Finally, we provide the fidelity 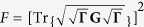 as a measure of the robustness of the reconstruction process via OC*m*T^52^, where **Γ** is the theoretical matrix and **G** is the measured matrix.

#### Beams with separable DoFs

**Figure 5:**
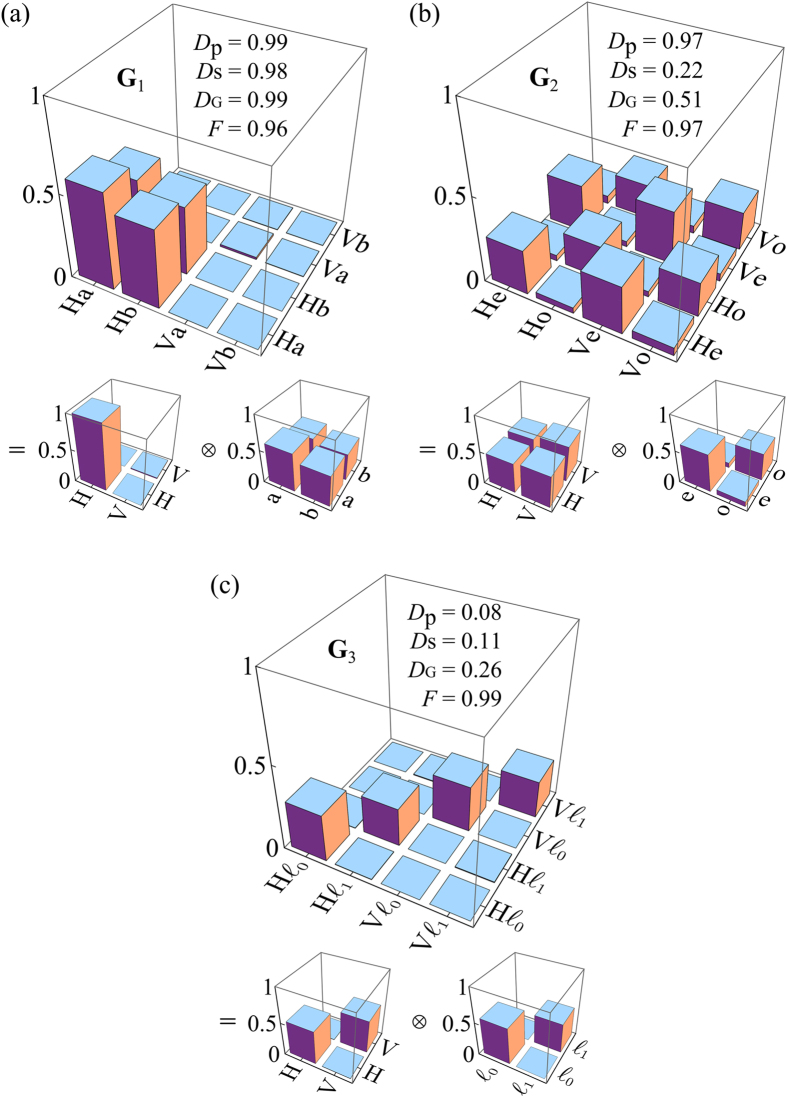
Measurements of **G** for beams having separable DoFs. (**a**) **G**_1_: both DoFs are coherent; (**b**) **G**_2_: polarization is coherent but the spatial DoF is incoherent; and (**c**) **G**_3_: both DoFs are incoherent. In each case, the reduced coherency matrices **G**_p_ and **G**_s_ are also depicted. The imaginary components are negligible, and are not shown.

We present in Fig. 5 three examples of beams having separable DoFs, 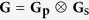. First, both polarization and spatial DoFs are coherent, 
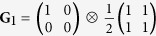
 [Fig. 5(a)]. Second, a hybrid beam in which polarization is pure (along D) but the beam is spatially incoherent, 
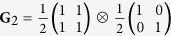
 [Fig. 5(b)]. Third, a completely incoherent beam 
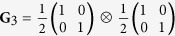
 [Fig. 5(c)]. In all three cases, the separability of the two DoFs is readily detected by visual inspection of **G** and confirmed by taking the direct product of the reduced coherency matrices.

#### Beams with non-separable DoFs

**Figure 6:**
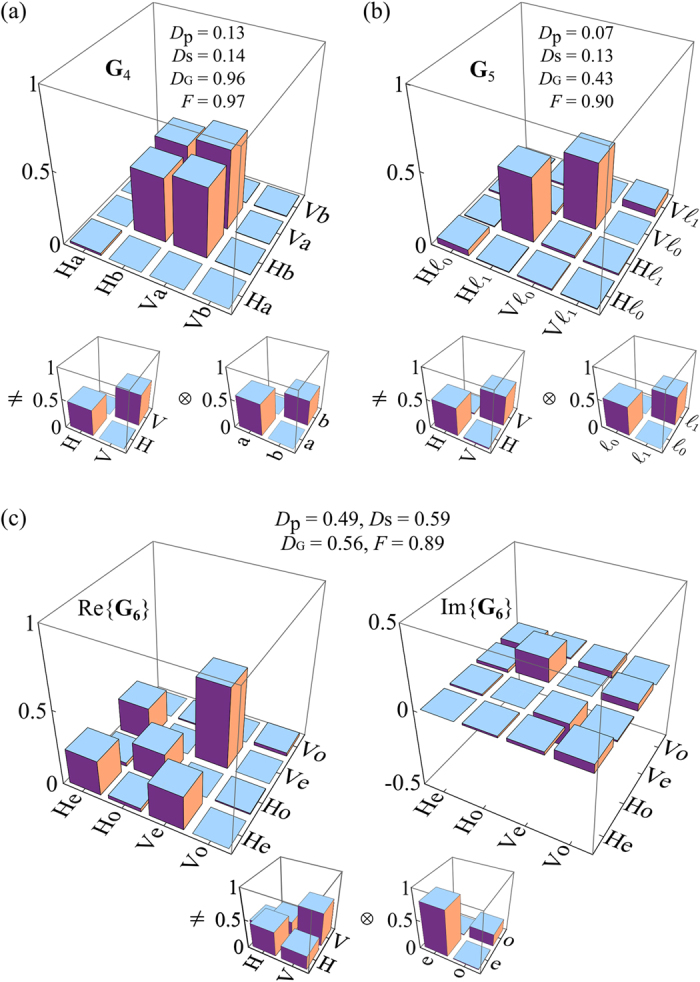
Measurements of **G** for beams having non-separable DoFs. (**a**) **G**_4_: classically entangled beam; and (**b**) **G**_5_: classically correlated beam. The imaginary components in both cases are negligible, and are not shown. (**c**) Measurement of the real and imaginary parts of **G**_6_: mixture of beams **G**_1_ and **G**_4_. In each case, the reduced coherency matrices **G**_p_ and **G**_s_ are also depicted.

We next present two fundamentally distinct classes of beams with non-separable **G** in Fig. 6(a,b). First, OC*m*T of a *classically entangled* beam **G**_4_ is shown in Fig. 6(a), wherein the beam is fully *coherent*, and yet the measures extracted from reduced coherency matrices indicate complete *in*coherence. In such a beam, the polarization and spatial modes occur in pairs—e.g., H with ‘a’ and V with ‘b’ (but never H with ‘b’ or V with ‘a’). In the traditional view of the double slit experiment, such coupling will produce no interference fringes, and the lack of visibility may be interpreted as the absence of spatial coherence, despite the beam being perfectly spatially coherent. This coupling between the DoFs is in fact encoded in the non-zero off-diagonal elements of **G** revealed once it is reconstructed through OC*m*T, but cannot be obtained from **G**_p_ or **G**_s_. Second, a *classically correlated* beam **G**_5_ is shown in Fig. 6(b) in which the same coupling between polarization and spatial modes occurs as in the previous example, except the different combinations are *incoherently* mixed and *not* linearly superposed. The partial global coherence—despite the complete lack of coherence for each DoF—is clear from the fact that not all the diagonal elements of **G**_5_ are equal as is the case in **G**_3_.

#### Mixture of beams with separable and non-separable DoFs

Finally, in Fig. 6(c) we depict **G**_6_ corresponding to a beam formed by statistically mixing the separable-coherent beam **G**_1_ and the classically entangled beam **G**_4_. The measurement of **G**_6_ indicates that part of the apparent incoherence in this beam stems from the intrinsic randomness in the individual DoFs, and part of it from the correlation, or classical entanglement, between the two DoFs.

## Discussion

The reconstruction of **G** allows for the unambiguous and complete mathematical expression of fields that are coherent, partially coherent, or incoherent, in either, or both, DoFs of an optical beam with two binary DoFs. The usefulness of this technique becomes specially apparent in cases where the DoFs are coupled or non-separable, and the traditional scalar measures of coherence provide a conflicting and fallacious account of beam coherence. The apparent absence of coherence in any DoF may be the result of intrinsic randomness due to statistical fluctuations, or due to the coupling or non-separability with another DoF. In the latter case, the measurement of **G** also provides the way for implementing unitary transformations required to undo such coupling, and restore coherence in the DoFs. The application of our work can be easily seen in the myriad applications of coherence under conditions of coupled DoFs, particularly those involving localized vector beams, sub-diffraction imaging, nanophotonics, and propagation through disordered media. Measurement of **G**, before and after transmission though a system that couples various DoFs, will help determine the characteristics of the system. This technique may hence find important applications in crystallography, atmospheric optics, and systems involving photonic crystals or anisotropic scatters, etc.

In summary, we have experimentally demonstrated for the first time a methodical, yet versatile, approach to reconstructing the 4 × 4 coherency matrix **G** of an optical beam having two binary DoFs, which we call *optical coherency matrix tomography*. We have explored three different physical realizations in which we combine polarization with spatial position, spatial parity, or orbital angular momentum modes. By exploiting the mathematical similarity with quantum state tomography of two photon states, we determine the minimal set of measurements required to reconstruct **G**. Although we have conducted the experiments for a beam with two binary DoFs, this methodology is equally applicable for a higher number of DoFs with *m*-ary levels each.

## Additional Information

**How to cite this article**: Kagalwala, K. H. *et al.* Optical coherency matrix tomography. *Sci. Rep.* **5**, 15333; doi: 10.1038/srep15333 (2015).

## Supplementary Material

Supplementary Information
