# Supplementary material for: Optical coherency matrix tomography
Source: Sci Rep. 2015 Oct 19;5:15333. doi: 10.1038/srep15333 (PMC4609991; doi:10.1038/srep15333)
Supplement: Supplementary Information [file srep15333-s1.pdf]

Supplementary Information for
Optical coherency matrix tomography

Kumel H. Kagalwala, H. Esat Kondakci, Ayman F. Abouraddy, and Bahaa E. A. Saleh

CREOL, The College of Optics & Photonics,

University of Central Florida, Orlando, Florida 32816, USA

(Dated: August 25, 2015)

Abstract

This document provides supplementary information for the manuscript "Optical coherency matrix tomography". We provide experimental details for generating beams for which the polarization is coupled with a spatial DoF. We also provide the complete set of experimental measurements for the coherency matrix **G** for polarization coupled with pairs of points, spatial parity modes, and orbital angular momentum modes.

In this supplementary document, we provide the complete results of three sets of experiments for optical coherency matrix tomography (OCmT). Each experiment probes a different spatial degree of freedom (DoF) of an optical beam in conjunction with polarization. In the main text, we presented selections from these three experiments, which are given here in their entirety. The results for the two-point Young's double slit case are given in Fig. S1, for the spatial parity modes in Fig. S2, and for the orbital angular momentum modes in Fig. S3.

POLARIZATION AND DOUBLE SLITS

The polarization and position-coupled beams in states $\mathbf{G}_1$ through $\mathbf{G}_6$ are prepared as follows:

(a) $\mathbf{G}_1$: the polarization and spatial DoFs are separable and both are coherent. This beam is produced by simply passing a horizontally polarized beam though the slits 'a' and 'b'.

(b) $\mathbf{G}_2$: the polarization DoF is coherent while the spatial DoF lacks coherence. This beam is produced by introducing spatial incoherence in a horizontally polarized beam, by using a diffuser, such as rotating ground glass, or a spatial light modulator (LCOS-SLM X10468-02, Hamamatsu Photonics K. K.).

(c) $\mathbf{G}_3$: both the polarization and spatial DoFs lack coherence. This beam is produced by first introducing spatial incoherence in the beam, and then passing it through a polarization scrambler (Liquid Crystal Polarization Rotator, Meadowlark Optics).

(d) $\mathbf{G}_4$: the polarization and spatial DoFs are classically entangled. This beam is produced by passing a vertically polarized beam through slit 'a', and a horizontally polarized beam through slit 'b'. The vertically polarized beam is obtained by placing a half-wave plate (HWP) at 45° at slit 'b'.

(e) $\mathbf{G}_5$: the polarization and spatial DoFs are classically correlated. This beam is produced by passing a vertically polarized beam through slit 'a', and a horizontally polarized beam through slit 'b', and then introducing spatial incoherence in the beam.

(f) $\mathbf{G}_6$: this beam is a mixture of the separable-coherent beam $\mathbf{G}_1$ and the classically entangled beam $\mathbf{G}_4$. It is produced by placing the polarization scrambler at slit 'b'.

POLARIZATION AND SPATIAL PARITY

The polarization and spatial parity-coupled beams in states $\mathbf{G}_1$ through $\mathbf{G}_6$ are prepared as follows:

(a) $\mathbf{G}_1$: the polarization and spatial DoFs are separable and both are coherent. This beam is produced by passing a diagonally polarized beam through the SLM having a zero phase step.

(b) $\mathbf{G}_2$: the polarization DoF is coherent while the spatial DoF lacks coherence. This beam is produced by first passing a horizontally polarized beam through the SLM. The phase pattern on the SLM switches periodically between the zero phase step and the π phase step. The beam then passes through a HWP at 22.5°.

(c) $\mathbf{G}_3$: both the polarization and spatial DoFs lack coherence. This beam is produced by passing it through the SLM whose phase pattern switches periodically between the zero phase step and the π phase step, and then scrambling the polarization.

(d) $\mathbf{G}_4$: the polarization and spatial DoFs are classically entangled. The beam is prepared by passing a diagonally polarized beam through the SLM having a π phase step.

(e) $\mathbf{G}_5$: the polarization and spatial DoFs are classically correlated. This beam is produced by scrambling the polarization and passing it through the SLM having a π phase step.

(f) $\mathbf{G}_6$: this beam is a mixture of the separable-coherent beam $\mathbf{G}_1$ and the classically entangled beam $\mathbf{G}_4$. It is produced by passing a diagonally polarized beam through the SLM whose phase pattern switches periodically between the zero phase step and the π phase step.

POLARIZATION AND OAM MODES

The polarization and OAM mode-coupled beams in states $\mathbf{G}_1$ through $\mathbf{G}_6$ are prepared as follows:

(a) $\mathbf{G}_1$: the polarization and spatial DoFs are separable and both are coherent. This beam is produced by passing a diagonally polarized beam through the SLM having a zero phase distribution.

(b) $\mathbf{G}_2$: the polarization DoF is coherent while the spatial DoF lacks coherence. This beam is produced by passing a horizontally polarized beam through the SLM. The phase pattern

on the SLM switches periodically between the zero phase distribution and the phase vortex $e^{i\phi}$, $0 \leq \phi < 2\pi$. The beam then passes through a HWP at 22.5°.

(c) $\mathbf{G}_3$: both the polarization and spatial DoFs lack coherence. This beam is produced by passing it through the SLM whose phase pattern switches periodically between the zero phase distribution and the phase vortex $e^{i\phi}$, $0 \leq \phi < 2\pi$, and then scrambling the polarization.

(d) $\mathbf{G}_4$: the polarization and spatial DoFs are classically entangled. The beam is prepared by passing a diagonally polarized beam through the SLM having the phase vortex $e^{i\phi}$, $0 \leq \phi < 2\pi$.

(e) $\mathbf{G}_5$: the polarization and spatial DoFs are classically correlated. This beam is produced by scrambling the polarization and passing it through the SLM having the phase vortex $e^{i\phi}$, $0 \leq \phi < 2\pi$.

(f) $\mathbf{G}_6$: this beam is a mixture of the separable-coherent beam $\mathbf{G}_1$ and the classically entangled beam $\mathbf{G}_4$. It is produced by passing a diagonally polarized beam through the SLM whose phase pattern switches periodically between the zero phase distribution and the phase vortex $e^{i\phi}$, $0 \leq \phi < 2\pi$.

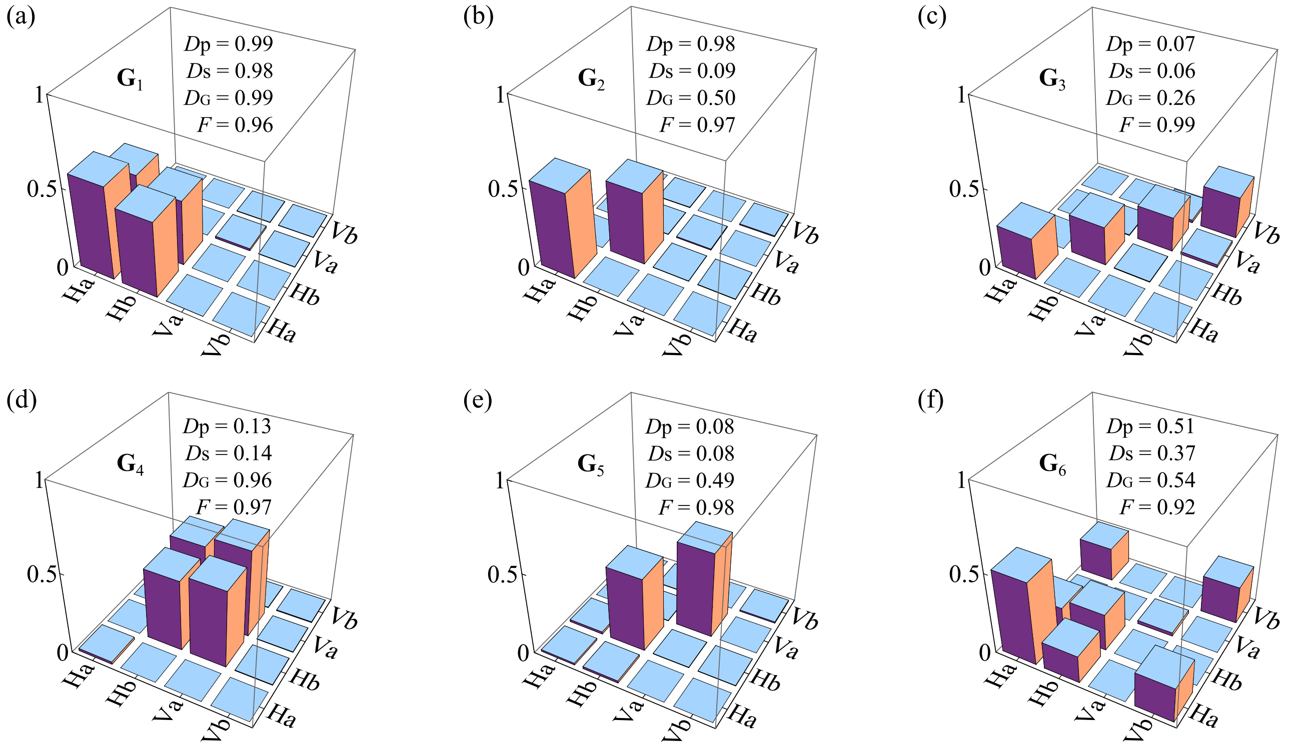

FIG. S1. Polarization and double slits. Measurements of $\mathbf{G}$ for beams having separable DoFs. (a) $\mathbf{G}_1$: both DoFs are coherent; (b) $\mathbf{G}_2$: polarization is coherent but the spatial DoF is incoherent; and (c) $\mathbf{G}_3$: both DoFs are incoherent. Measurements of $\mathbf{G}$ for beams having non-separable DoFs. (a) $\mathbf{G}_4$: classically entangled beam; (b) $\mathbf{G}_5$: classically correlated beam; and (c) $\mathbf{G}_6$: mixture of beams $\mathbf{G}_1$ and $\mathbf{G}_4$. The imaginary components in all cases are negligible, and are not shown.

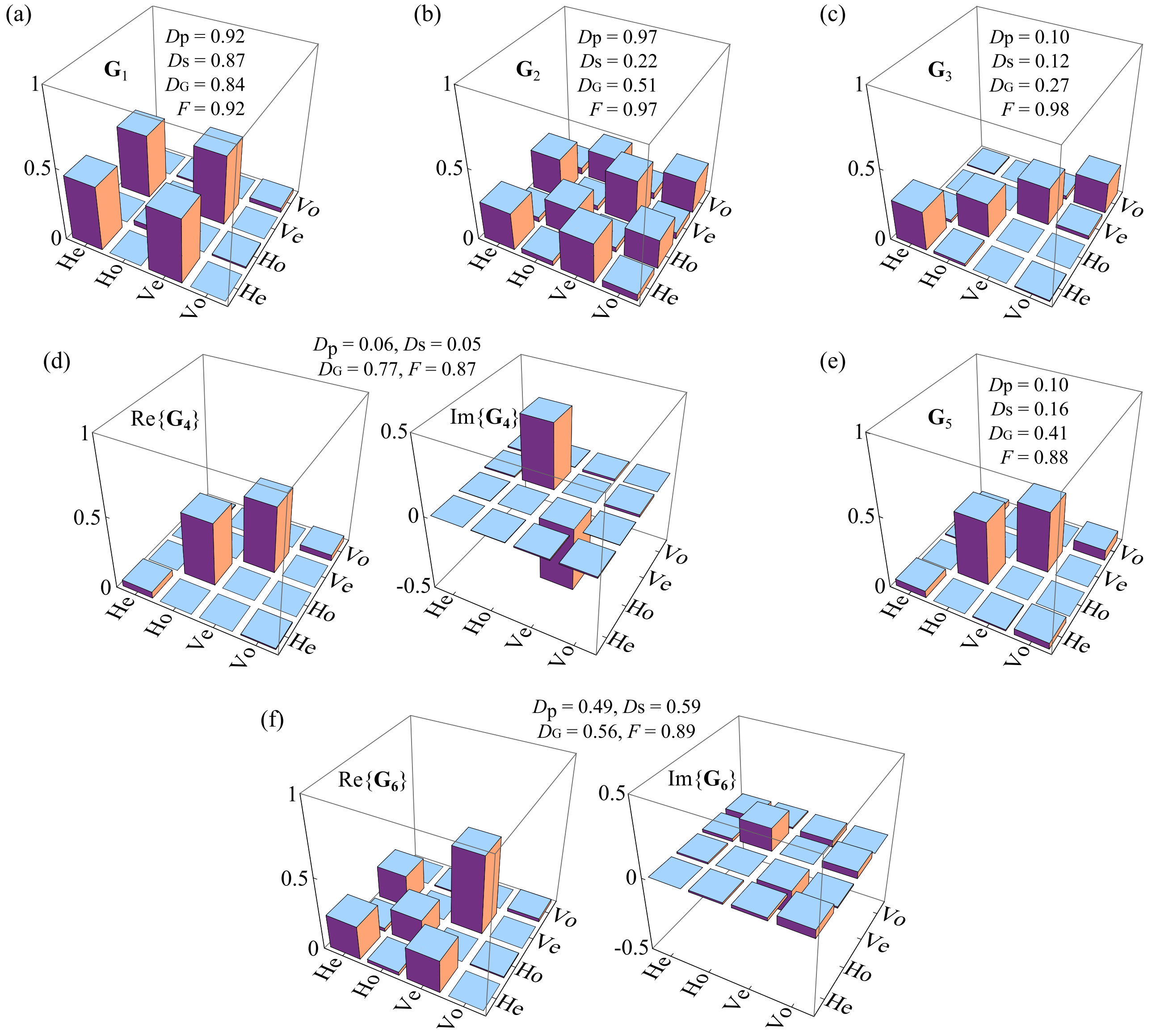

FIG. S2. Polarization and spatial parity. Measurements of $\mathbf{G}$ for beams having separable DoFs. (a) $\mathbf{G}_1$: both DoFs are coherent; (b) $\mathbf{G}_2$: polarization is coherent but the spatial DoF is incoherent; and (c) $\mathbf{G}_3$: both DoFs are incoherent. The imaginary components in these cases are negligible, and are not shown. Measurements for the real and imaginary parts of $\mathbf{G}$ for beams having non-separable DoFs. (a) $\mathbf{G}_4$: classically entangled beam; (b) $\mathbf{G}_5$: classically correlated beam; and (c) $\mathbf{G}_6$: mixture of beams $\mathbf{G}_1$ and $\mathbf{G}_4$.

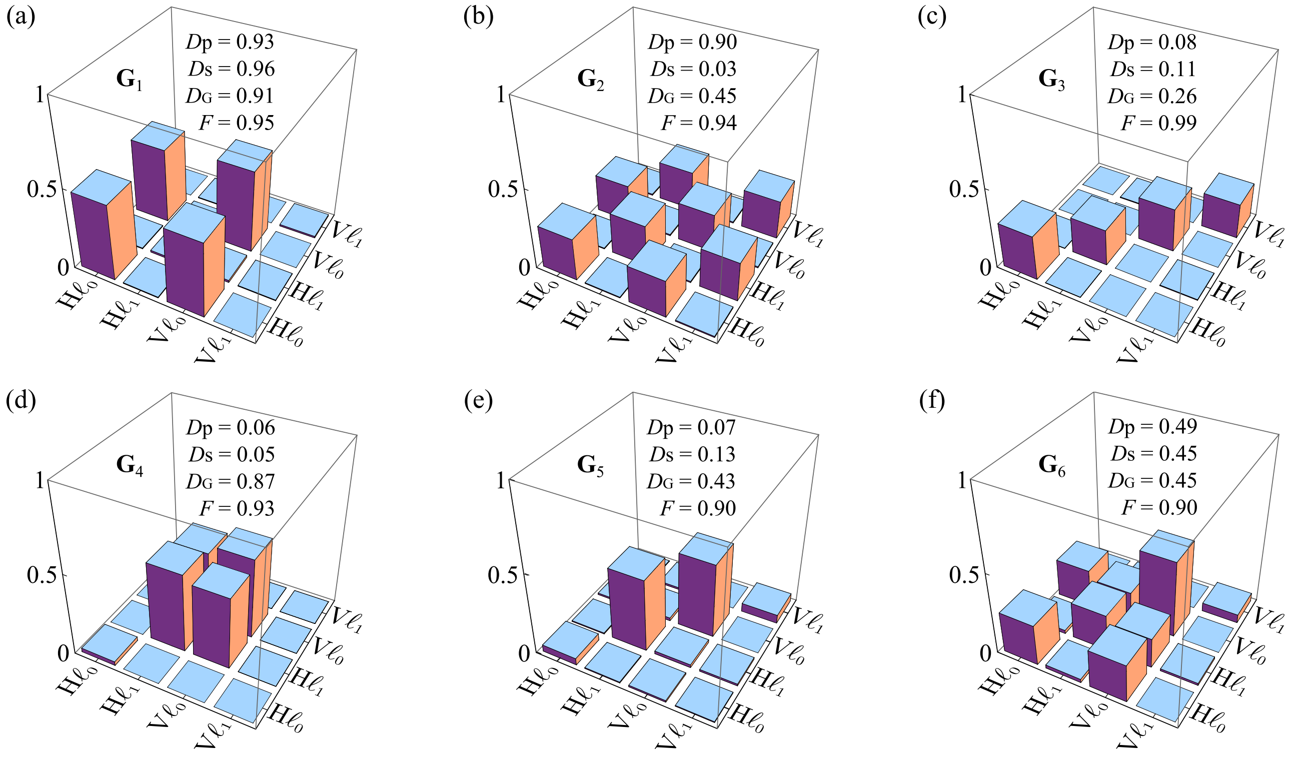

FIG. S3. Polarization and OAM modes. Measurements of $\mathbf{G}$ for beams having separable DoFs. (a) $\mathbf{G}_1$: both DoFs are coherent; (b) $\mathbf{G}_2$: polarization is coherent but the spatial DoF is incoherent; and (c) $\mathbf{G}_3$: both DoFs are incoherent. Measurements of $\mathbf{G}$ for beams having non-separable DoFs. (a) $\mathbf{G}_4$: classically entangled beam; (b) $\mathbf{G}_5$: classically correlated beam; and (c) $\mathbf{G}_6$: mixture of beams $\mathbf{G}_1$ and $\mathbf{G}_4$. The imaginary components in all cases are negligible, and are not shown.
